# Exploring the mechanism and phytochemicals in Psoraleae Fructus-induced hepatotoxicity based on RNA-seq, in vitro screening and molecular docking

**DOI:** 10.1038/s41598-023-50454-0

**Published:** 2024-01-19

**Authors:** Huiying Shang, Xian Liu, Jinchao Pan, Hongbo Cheng, Zengchun Ma, Chengrong Xiao, Yue Gao

**Affiliations:** 1 Tianjin University of Traditional Chinese Medicine, Tianjin, 301617 People’s Republic of China; 2 Department of Pharmaceutical Sciences, Beijing Institute of Radiation Medicine, Beijing, 100850 People’s Republic of China; 3 Faculty of Environment and Life Science, Beijing University of Technology, Beijing, 100124 People’s Republic of China; 4 School of Pharmacy, Guangdong Pharmaceutical University, Guangzhou, 510006 People’s Republic of China

**Keywords:** Computational biology and bioinformatics, Molecular biology

## Abstract

Psoraleae Fructus (PF) is a widely-used herb with diverse pharmacological activities, while its related hepatic injuries have aroused public concerns. In this work, a systematic approach based on RNA sequencing (RNA-seq), high-content screening (HCS) and molecular docking was developed to investigate the potential mechanism and identify major phytochemicals contributed to PF-induced hepatotoxicity. Animal experiments proved oral administration of PF water extracts disturbed lipid metabolism and promoted hepatic injuries by suppressing fatty acid and cholesterol catabolism. RNA-seq combined with KEGG enrichment analysis identified mitochondrial oxidative phosphorylation (OXPHOS) as the potential key pathway. Further experiments validated PF caused mitochondrial structure damage, mtDNA depletion and inhibited expressions of genes engaged in OXPHOS. By detecting mitochondrial membrane potential and mitochondrial superoxide, HCS identified bavachin, isobavachalcone, bakuchiol and psoralidin as most potent mitotoxic compounds in PF. Moreover, molecular docking confirmed the potential binding patterns and strong binding affinity of the critical compounds with mitochondrial respiratory complex. This study unveiled the underlying mechanism and phytochemicals in PF-induced liver injuries from the view of mitochondrial dysfunction.

## Introduction

Psoraleae Fructus (also termed as “Bu-Gu-Zhi” in Chinese, PF), the dried matured seeds of the leguminous plant *Psoralea corylifolia* L., is believed to show good therapeutic effect of warming and tonifying the Kidney-Yang, gathering spirit and enriching the bone marrow, according to the statement in Compendium of Materia Medica^1^. PF has been extensively used to treat osteoporosis, arthralgia, vitiligo and other diseases in Asia for a long history^2–4^. Modern pharmacological researches also manifested PF had anti-tumor, antioxidant, antimicrobial, anti-inflammatory and anti-depressive activities^5–7^. With the prevalence of herbal decoctions containing PF, the adverse events were frequently reported^8–10^. Among 2665 cases of adverse reactions after taking PF preparations monitored by China's Food and Drug Administration from 2004 to 2016, the incidence rate of hepatic injuries ranked the highest^11^. Therefore, investigations on the latent mechanism and hepatotoxic phytochemicals are of great significance to ensure safe and rational utilization of PF in clinical practice.

To date, more than 180 phytochemicals have been isolated and identified from PF, including flavonoids, coumarins, monoterpenoid phenols and benzofurans^12^. Traditional toxicological experiments seem unimaginable to realize the rapid and high-throughput screening of hepatotoxic compounds from the large compound libraries. Previous studies evaluated hepatotoxicities of few phytochemicals in PF by detecting the level of reactive oxygen species (ROS), nuclear area or biochemical indicators^13^. Considering diverse kinds of skeleton structure, the screening scope of phytochemicals should be extended to provide more comprehensive information about structure–activity relationship. Additionally, exploring novel screening methods based on the key mechanism will improve the accuracy of toxicants prediction.

Mitochondrial toxicity was the main reason for clinical failure of candidate compounds and drugs^14^. Mitochondria utilize the substrates generated from tricarboxylic acid (TCA) cycle to enter electron transport chain (ETC) for oxidative phosphorylation (OXPHOS), thus realizing ATP biosynthesis^15^. OXPHOS impairment increases mitochondrial ROS production, limited ATP generation and caused metabolism disorder^16^. Accumulating researches proved that OXPHOS impairment played a vital role in the pathogenesis of drug-induced liver injury^17^. However, the potential relationship between mitochondrial OXPHOS and PF-induced hepatotoxicity is still under interpretation. Further screening of mitototoxic phytochemicals in PF is in urgent need to facilitate the study on PF detoxification through processing or in combination with other drugs.

**Figure 1:**
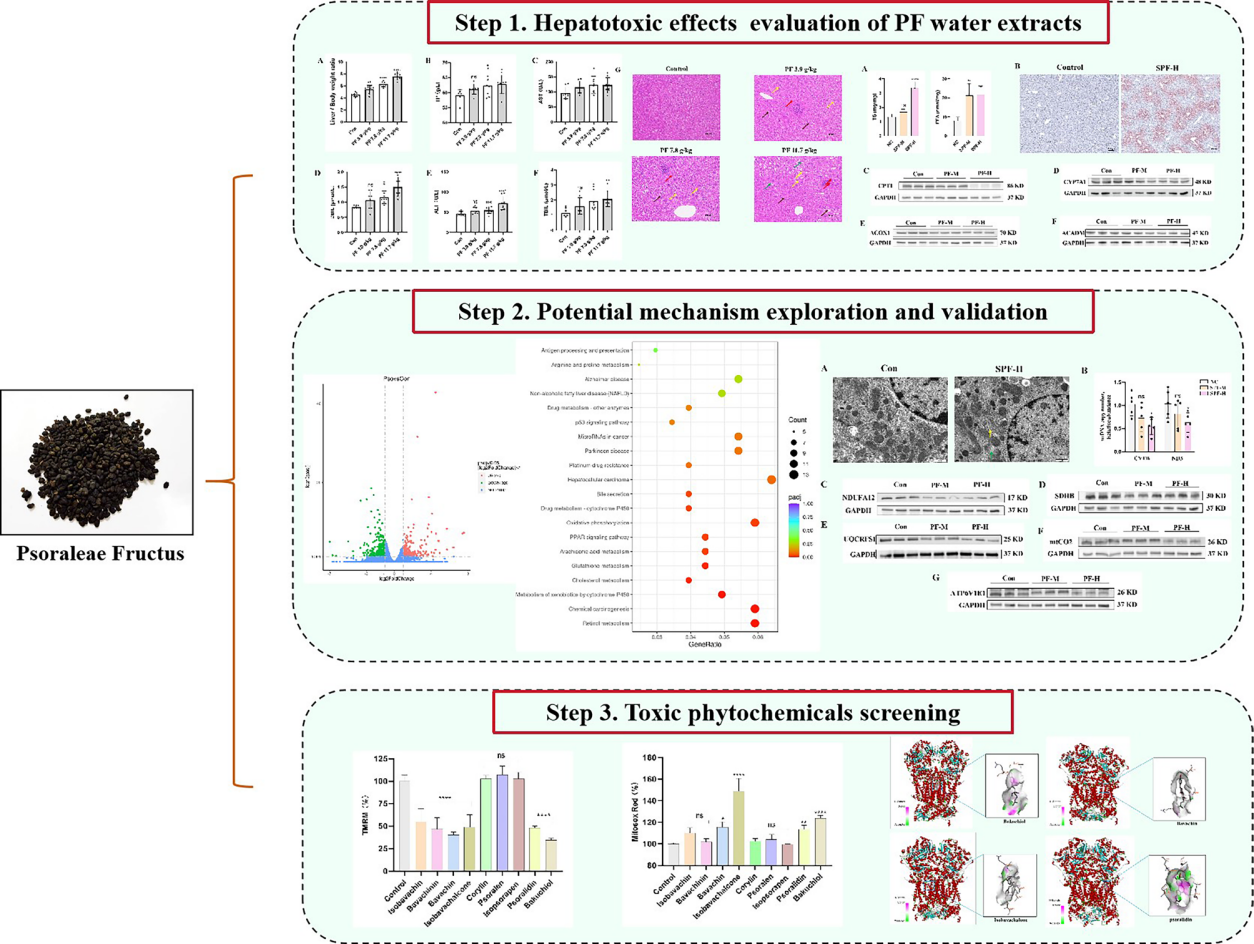
Schematic diagram of strategies for clarifying the potential mechanism and toxic phytochemicals underlying PF-induced hepatotoxicity.

In this study, the integrated approach based on RNA sequencing (RNA-seq), in vitro screening and molecular docking was established to explore the mechanism and identify toxic constituents in PF (Fig. 1). The hepatotoxic effects were firstly demonstrated by elevated hepatic biochemical indexes, histopathological changes and hepatic lipid accumulation in mice. RNA-seq combined with KEGG enrichment analysis and experimental validations indicated OXPHOS impairment as the key molecular mechanism. Based on mitochondrial dysfunction, hepatotoxic constituents were screened by high-content screening (HCS) analysis. Finally, the strong binding affinity and binding patterns of bavachin, isobavachalcone, bakuchiol and psoralidin with mitochondrial respiratory complex III were confirmed by molecular docking.

## Materials and methods

### TCM, chemicals and reagents

Salt-processed Psoraleae Fructus (SPF) samples were purchased from Beijing LVYE Pharmaceutical Co. Ltd (Beijing, China). Psoralen (B20123), isopsoralen (B21515), bavachinin (B20120), psoralidin (B20118), bakuchiol (B20121) and isobavachalcone (B21513) were purchased from Shanghai YuanYe Bio-Technology Co. Ltd (Shanghai, China). Corylin (T6S0141) and Cell Counting Kit-8 (CCK-8, C0005) were bought from Topscience Co. Ltd (Shanghai, China). Isobavachin (SI8110) was acquired from Solarbio Life Science (Beijing, China). Bavachin (E031951) was obtained from EFEBIO (Shanghai, China). Hoechst 33,342 (14,533) were purchased from Sigma-Aldrich (St. Louis, MO, United States). Tetramethylrhodamine Methyl Ester Perchlorate (TMRM, T3608) was obtained from Tokyo Chemical Industry (Shanghai, China). MitoSOX™ Red mitochondrial superoxide indicator (M36008) was purchased from Thermo Fisher Scientific (Waltham, MA, United States). Anti-CYP7A1 rabbit polyclonal antibody (510,615), Anti-ACOX1 rabbit monoclonal antibody (R23371), anti-ACADM rabbit monoclonal antibody (R383273), anti-SDHB rabbit monoclonal antibody (R381845) and mtCO_2_ rabbit monoclonal antibody (R25027) were bought from Zen Bio (Chengdu, Sichuan, China). Anti-CPT1 rabbit monoclonal antibody (15184-1-AP) and anti-ATP6VE1 rabbit polyclonal antibody (15280-1-AP) were purchased from Proteintech (Wuhan, Hubei, China). Anti-UQCRFS1 rabbit monoclonal antibody (ab191078), anti-NDUFA12 rabbit monoclonal antibody (ab192517) and anti-GAPDH rabbit monoclonal antibody (ab181602) were purchased from Abcam (Cambridge, MA). Triglyceride (TG) content assay kit (BC0620) and free fatty acids (FFA) content assay kit (BC0590) were purchased from Solarbio Life Science (Beijing, China).

### Preparation of PF water extracts

SPF was soaked in a sixfold amount of water overnight at room temperature and refluxed twice for 2 h each. The PF water extracts were filtered, mixed and concentrated under reduced pressure at 65 °C. The resulting concentrated liquid was frozen into powder by the vacuum freeze dryer (Song Yuan, Beijing, China). The yield of PF water extract was 21% and the resulting samples were stored at 4 °C.

### Animals

40 Kunming mice (20 female mice and 20 male mice) with body weights of 22–25 g were purchased from Beijing Vital River Laboratory Animal Technology Co. Ltd. All experimental procedures and animal care were approved by the Ethics Committee of Beijing Institute of Radiation Medicine (Approval No. IACUC-DWZX-2022-865). All animals received research according to the criteria outlined in the “Guide for the Care and Use of Laboratory Animals” prepared by the National Academy of Sciences and published by the National Institutes of Health. All methods were reported in accordance with ARRIVE guidelines. All mice were housed at 20–25 °C and 55% relative humidity environment using a 12 h light/dark cycle and had free access to food and water. After acclimatization to the environment for a week, the mice were randomly divided into four groups (sex in half, *n* = 10): a control group, low dose group (3.9 g/kg), middle dose group (7.8 g/kg) and high dose group (11.7 g/kg). The doses are equivalent to 3, 6 and 9 times of a typical human dose (10 g/70 kg/day), respectively. The PF extracts were resuspended in ultrapure water and administered intragastrically for 4 weeks. The control group was administered with an equal volume of ultrapure water. After cervical dislocation was used to sacrifice all of the mice, the livers were harvested, cleaned with saline and used for the next experiments.

### Blood biochemistry

Serum samples were obtained by centrifuging the blood samples at 4000 rpm for 15 min at 4 °C. Serum levels of alanine transaminase (ALT), aspartate transaminase (AST), total protein (TP), total bilirubin (TBIL), and direct bilirubin (DBIL) were detected using the automatic blood biochemistry analysis instrument (Roche, cobas c311).

### Histopathology

The liver samples were weighed and preserved in 4% formalin. Organ coefficient [(organ weight × 100)/body weight] were calculated. The livers were embedded in paraffin, sectioned and stained with hematoxylin and eosin (H&E) or red oil.

### Determination of hepatic TG and FFA concentrations

Hepatic TG and FFA concentrations were determined respectively using TG and FFA content assay kit according to the manufacturer’s instructions. Optical density (OD) values for TG and FFA were measured with live cell imaging system (Cytation 5, BioTek, USA) at 420 and 550 nm, respectively.

### RNA sequencing and data analysis

The liver samples from the control and high dose groups (four biological replicates per condition) were rapidly quenched in liquid nitrogen and stored at − 80 °C. A portion of the liver samples were sent to Novogene Corporation (Beijing, China) for library construction and sequencing. RNA is extracted from liver tissues using standard extraction methods, followed by rigorous quality control of the RNA samples using an Agilent 2100 bioanalyzer. After library construction, initial quantification was performed using a Qubit 2.0 Fluorometer, followed by detection of the insert size of the library using an Agilent 2100 bioanalyzer and accurate quantification of the effective library concentration by qRT-PCR. In the step of quality data control, clean data were obtained by removing reads containing adapter, reads containing N base, and low-quality reads from raw data. All the downstream analyses were based on clean data with high quality. Differential expression analysis of two conditions/groups was performed using the DESeq2 R package. DESeq2 provides statistical routines for determining differential expression in digital gene expression data using a model based on the negative binomial distribution. Kyoto Encyclopedia of Genes and Genomes (KEGG) pathway enrichment analysis (*p*-value < 0.05) were performed using clusterProfiler software. KEGG is a comprehensive database that integrates genomic, chemical and phylogenetic functional information^18–20^.

### Protein extraction and western blot

Liver proteins were collected using RIPA lysis buffer (C1053, Applygen Technologies, Beijing, China) containing protease/phosphatase inhibitor cocktail (GRF101/102, EpiZyme, Shanghai, China). Protein concentrations were determined using the BCA protein assay reagent kit (P1511, Applygen Technologies, Beijing, China). Protein samples were separated by SDS-PAGE gels and transferred onto a polyvinylidene fluoride membranes (Millipore, Billerica, MA, United States). After blocked with 5% nonfat dry milk (PS112L, Epizyme, Shanghai, China) overnight at 4 °C, the membranes were incubated successively with specific primary and secondary antibodies. The protein blots were visualized with Omni-ECL™ Femto Light Chemiluminescence Kit (SQ201, Epizyme, Shanghai, China) and automatic exposure system (Image Quant LAS500, GE, Fairfield, CT, USA).

### Quantification of mtDNA copy number

Total DNA was extracted from liver tissues as previously reported^21^. Briefly, DNA was extracted using lysis buffer (10 mM Tris–HCl, pH 8.0, 150 mM NaCl, 20 mM EDTA, 1% SDS, and 0.2 mg/ml Proteinase K) and incubated at 55 °C for 6 h. After extraction by phenol–chloroform system and precipitation in isopropanol, DNA was resuspended in TE buffer. PCR was performed in a reaction volume of 20 μL, with 10 μL of Hieff UNICON® Universal Blue qPCR SYBR Green Master Mix (11184ES08, Yeasen, Shanghai, China), 0.4 μL of 10 μM forward and reverse primers, 0.1 μL of 25 ng DNA and 9.1 μL of nuclease-free water. The mtDNA copy number was determined by comparing mitochondrial genomes (CYTB and ND3) with nuclear genomes (Actin) using Bio-Rad (CFX96, USA). Primers are listed in Table 1.

**Table 1:** The primer sequence of genes for qPCR.

Gene	Primer (5′-3′)	Length
CYTB	F: ATTGGAACAACCCTAGTCGAATGAA	25
	R: GATTGCTAGGGCCGCGATAA	20
ND3	F: TCCATATGAATGCGGATTTGAC	22
	R: TTGCTCATGGTAGTGGAAGTAGAAG	25
Actin	F: CTCCAGAACGCAAGTACTCT	20
	R: CCAGCTTCGTCGTATTCCTG	20

### Cell culture

AML-12 cells were purchased from Procell (CL-0602, Wuhan, China) and cultured in cultured in DMEM/F12 medium (Gibco, Thermo Fisher Scientific, USA) with the supplement of 10% fetal bovine serum (FBS, Biological Industrials) and 1% penicillin/streptomycin (FG101-01, Transgene, China). AML-12 cells were maintained in a humidified incubator containing 5% CO_2_ at 37 °C.

### High-content screening analysis

AML-12 cells were seeded into 96-well plates and incubated at 37 °C in an atmosphere containing 5% CO_2_ for 12 h until they visibly reached confluence. After the cells were treated with the same concentration of each drug (15 μmol/L) for 24 h, the supernatants were removed from the cell plates and the fluorescent dyes were added. Hoechst 33,342 was applied to characterize cell counts and nuclear area. TMRM and MitoSOX™ Red were applied to characterize mitochondrial membrane potential (MMP) and mitochondrial superoxides, respectively. Then, the multi-parameter cytotoxicities were measured by a high content imaging system (Molecular Devices, Metaxpress) and subsequent data analysis was performed by Harmony 3.0 Software (Perkin Elmer, Waltham, MA, USA). Each experiment was assayed in triplicate wells and three independent replicates.

#### Molecular docking

The 3D structures of bavachin, isobavachalcone, bakuchiol and psoralidin were drawn by ChemBioDraw Ultra 20.0 and then subjected to energy optimization by the MM2 force field. The 3D structure of mitochondrial respiratory complex III (PDB ID: 5XTE) was downloaded from the PDB (http://www.rcsb.org/pdb/home/home.do)^22,23^. The ligand and protein files were prepared using AutoDock tools. The Protein was optimized by removing water molecules, adding hydrogen, adding Geister charges, and protein file was saved in PDBQT format. Ligand file was also saved in PDBQT format. The protein–Ligand Grid map was calculated surrounding active site of protein molecule. Molecular docking analysis were carried out by Autodock Vina. Autodock Vina was run using configuration file and output file generated contained theoretical binding affinity. The lesser binding affinity corresponds to better results. The docking results were visualized using PyMol.

### Statistical analysis

All results were presented as mean ± standard deviation (SD) and generated from at least three independent experiments. One-way ANOVA test was employed for the comparisons among multiple groups and student’s *t* test was used for the comparison between two groups. A value of *p* < 0.05 was chosen as the threshold for statistical significance. Statistical analysis was performed using GraphPad Prism version 8 (GraphPad Software, La Jolla, CA).

### Ethics approval

Every effort was devoted to minimizing pain and discomfort caused to the animals. This study was approved by the Ethics Committee of the Beijing Institute of Radiation Medicine (Approval No. IACUC-DWZX-2022-865). All animal experiments were the criteria outlined in the “Guide for the Care and Use of Laboratory Animals” prepared by the National Academy of Sciences and published by the National Institutes of Health. All methods were reported in accordance with ARRIVE guidelines and ICLAS ethical guidelines.

## Results

### PF led to impaired liver function and histopathological injuries

**Figure 2:**
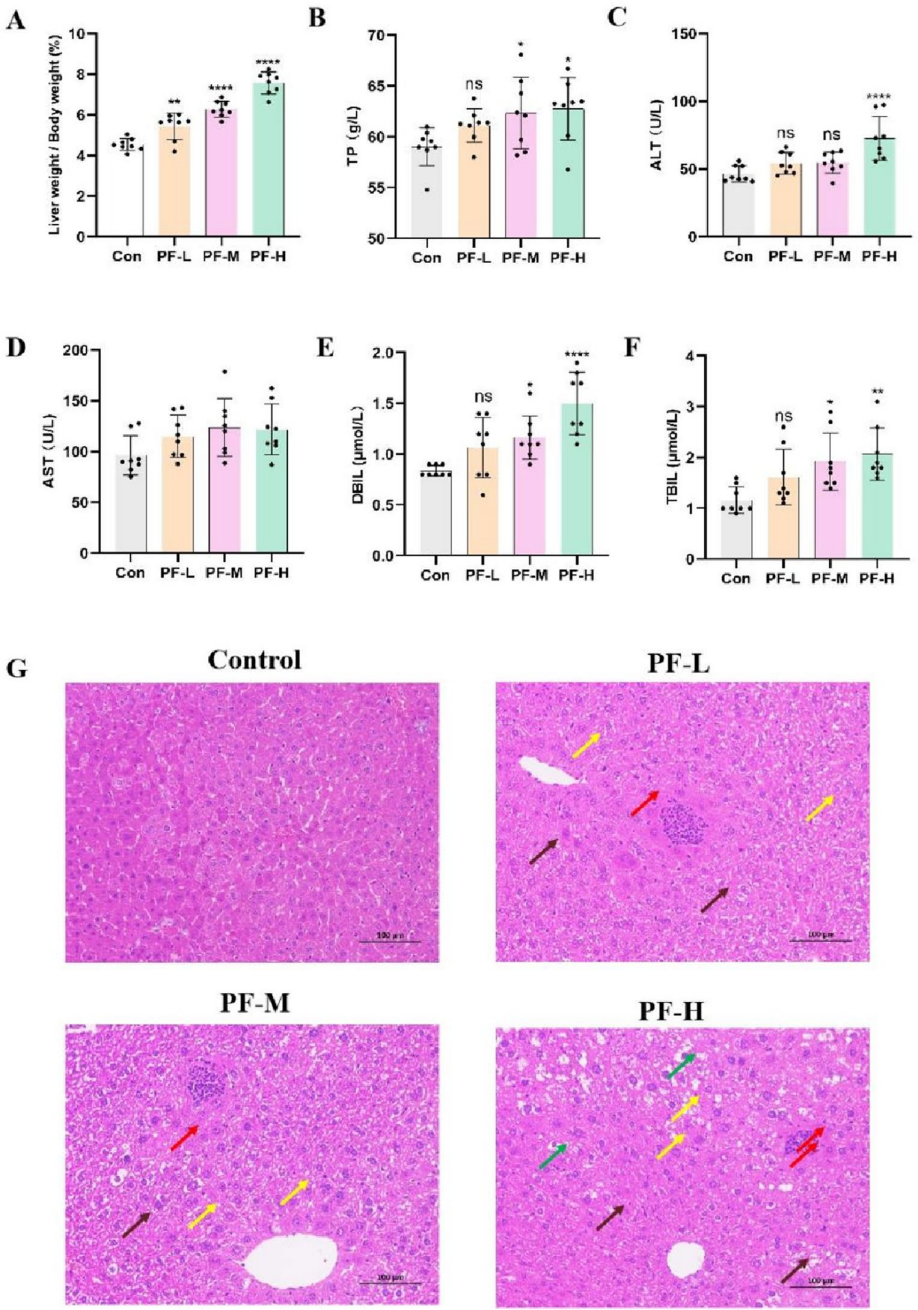
PF led to impaired liver function and histopathological injuries. (**A**) The liver to body weight ratio after treatment with PF water extracts. (**B**–**F**) Blood biochemical indicators for liver function after administration of PF water extracts. (**G**) Representative histopathological microphotographs of livers samples (H&E staining). Yellow arrows indicate cytoplasmic microvesicle formation, brown arrows indicate eosinophilic granules, red arrows indicate inflammatory infiltration and green arrows indicate cytoplasmic vacuolization. ns, no significant; *p < 0.05, **p < 0.01, ****p < 0.0001 versus the control group.

To investigate the hepatotoxicity of PF water extracts, the liver to body weight ratio were calculated for each group. The liver coefficients of PF-treated groups were obviously higher than the control group (Fig. 2A). Serum levels of TP, ALT, AST, DBIL and TBIL increased significantly in a dose-dependent manner after PF treatment (Fig. 2B–F). The morphological feature of liver tissue is direct and critical evidence for the diagnosis of liver damage. As shown in Fig. 2G, histopathological examination of the liver samples from PF-treated groups displayed varying degrees of hepatocytes swelling, cytoplasm rarefaction, hepatic steatosis, inflammatory infiltration inside the liver lobules, and accumulation of eosinophilic granules when in comparison with the control group.

### PF promoted lipid metabolism disorder by inhibiting fatty acids β-oxidation and cholesterol catabolism

**Figure 3:**
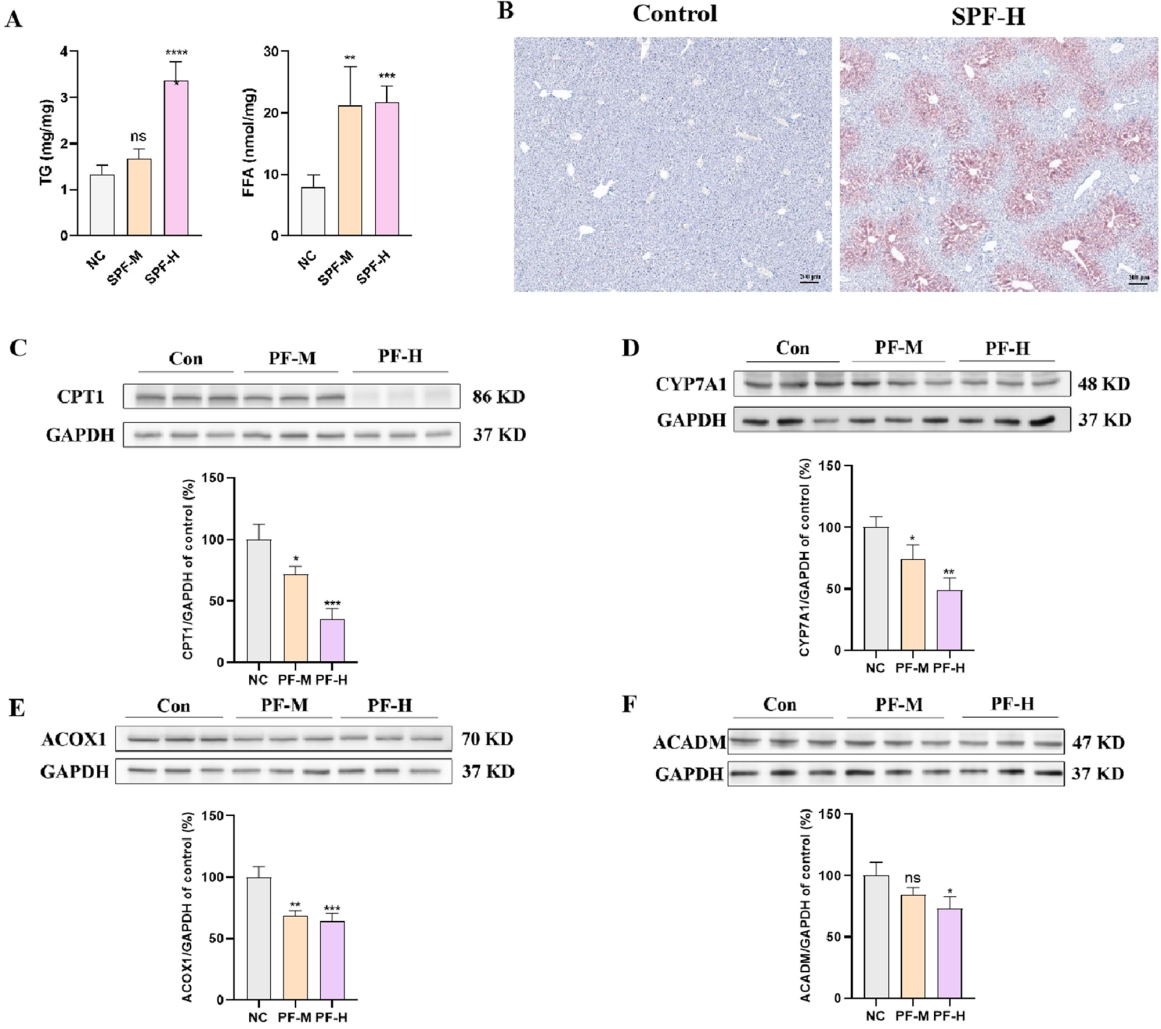
PF promoted lipid metabolism disorder by inhibiting fatty acids β-oxidation and cholesterol catabolism. (**A**) Hepatic TG and FFA contents. (**B**) Red oil staining of liver sections. (**C**–**F**) Western blots detect the expressions of CPT1, CYP7A1, ACOX1 and ACADM; These protein levels normalized to GAPDH were quantified by Image J. ns, no significant; *p < 0.05, **p < 0.01, ***p < 0.01 versus the control group.

Lipid metabolism disorder is the early stage of DILI and accelerate the progress of hepatic injuries to more severe inflammation and fibrosis^24^. Indicated by H&E staining of liver tissues, hepatic TG and FFA contents were measured. As shown in Fig. 3A, hepatic TG and FFA contents in PF group increased dose-dependently. Moreover, red oil staining showed that PF promoted the accumulation of lipid droplet in liver tissues (Fig. 3B). Carnitine palmitoyltransferase 1 (CPT1) is the rate-miting enzyme in the transport of long-chain fatty acids across mitochondrial membrane and its deficiency hampered fatty acids β-oxidation^25^. Acyl-coenzyme A oxidase 1 (ACOX1) and medium-chain specific acyl-CoA dehydrogenase (ACADM) initiated β-oxidation by breaking down fatty acids to acyl-CoAs^26,27^. Cytochrome P450 7A1 (CYP7A1) is the vital enzyme in the conversion of cholesterol to bile acids^28^. Western blots showed that PF potently suppressed the expressions of these genes (Fig. 3C–F), which might result in hepatic lipid accumulation.

### RNA-seq combined with enrichment analysis unveiled PF-induced hepatotoxicity is associated with mitochondrial OXPHOS

**Figure 4:**
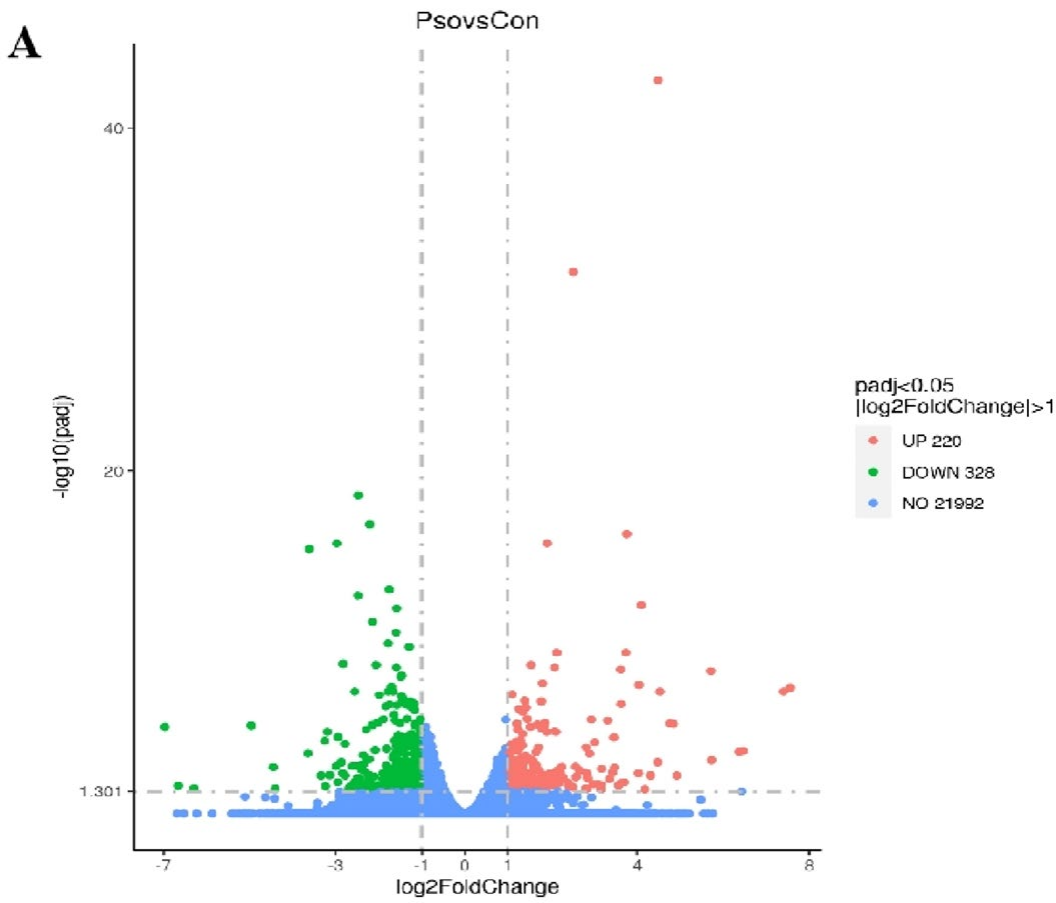
Volcano plots. (**A**) DEGs based on the criteria of |log2(FC)|> 1 and *p*-value < 0.05.

**Figure 5:**
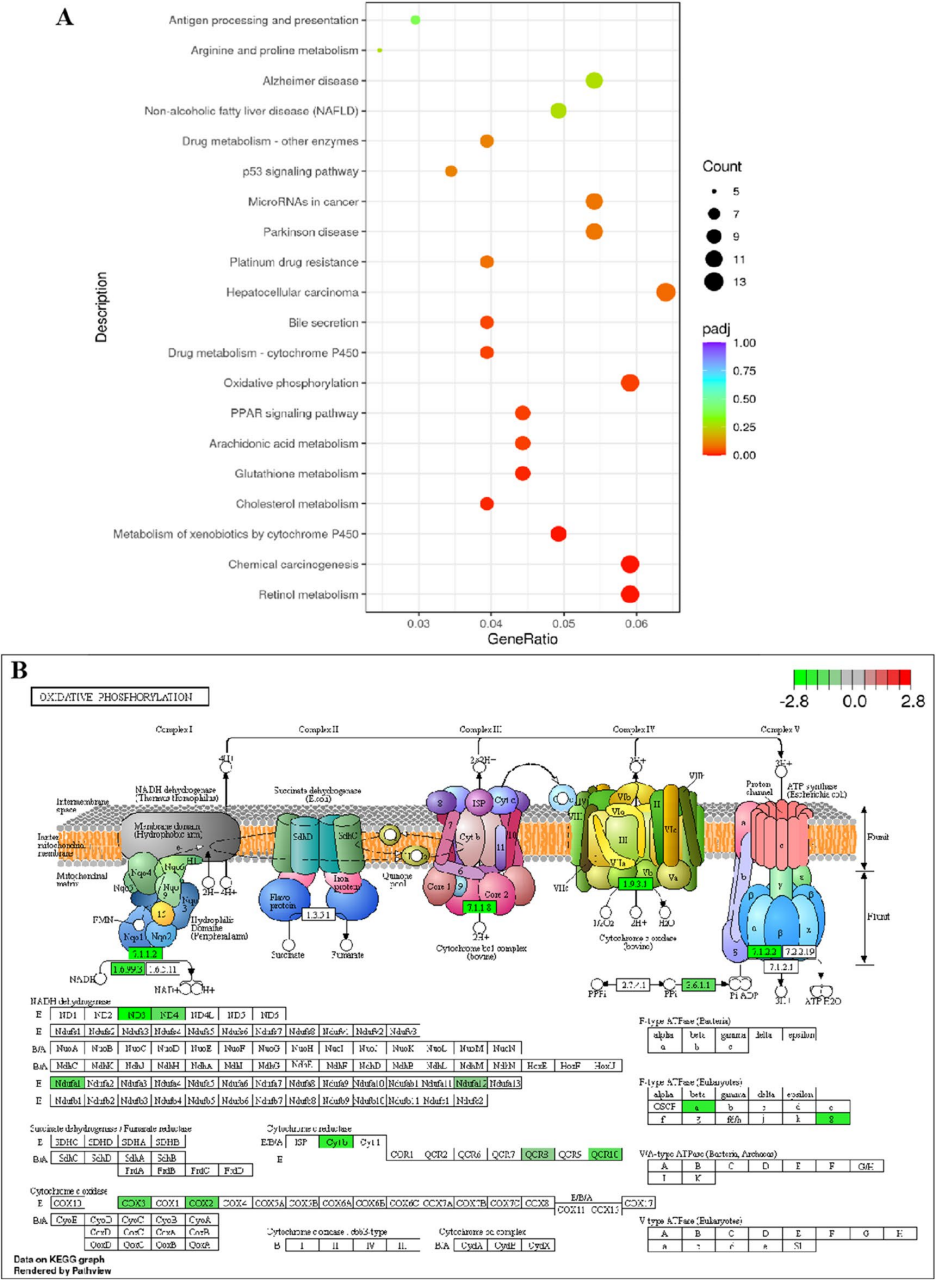
KEGG enrichment analysis of DEGs in PF group. (**A**) The top 20 enriched KEGG pathways of DEGs. (**B**) The KEGG enrichment results of DEGs in OXPHOS pathway.

Transcriptomic analysis was performed to gain an overview of the latent mechanism underlying PF-induced hepatotoxicity. As shown in the volcano plot (Fig. 4), there were a total of 548 differentially expressed genes (DEGs) (|Fold change|> 2, *p*-value < 0.05, 220 upregulated genes and 328 downregulated genes) in PF group. KEGG enrichment analysis were conducted to explore the physiological and pathological significance of the DEGs. KEGG results showed that DEGs mainly enriched in the pathways including OXPHOS, hepatocellular carcinoma, retinol metabolism and metabolism of xenobiotics by CYP450 enzymes (Fig. 5A). As the major manner of cellular energy supplement, OXPHOS aroused our interests. Further details were shown in KEGG pathway map. Down-regulated DEGs were critical components of complex I, III, IV and V in electron transfer chain (Fig. 5B).

### PF induced hepatic mitochondrial injury by inhibiting OXPHOS.

**Figure 6:**
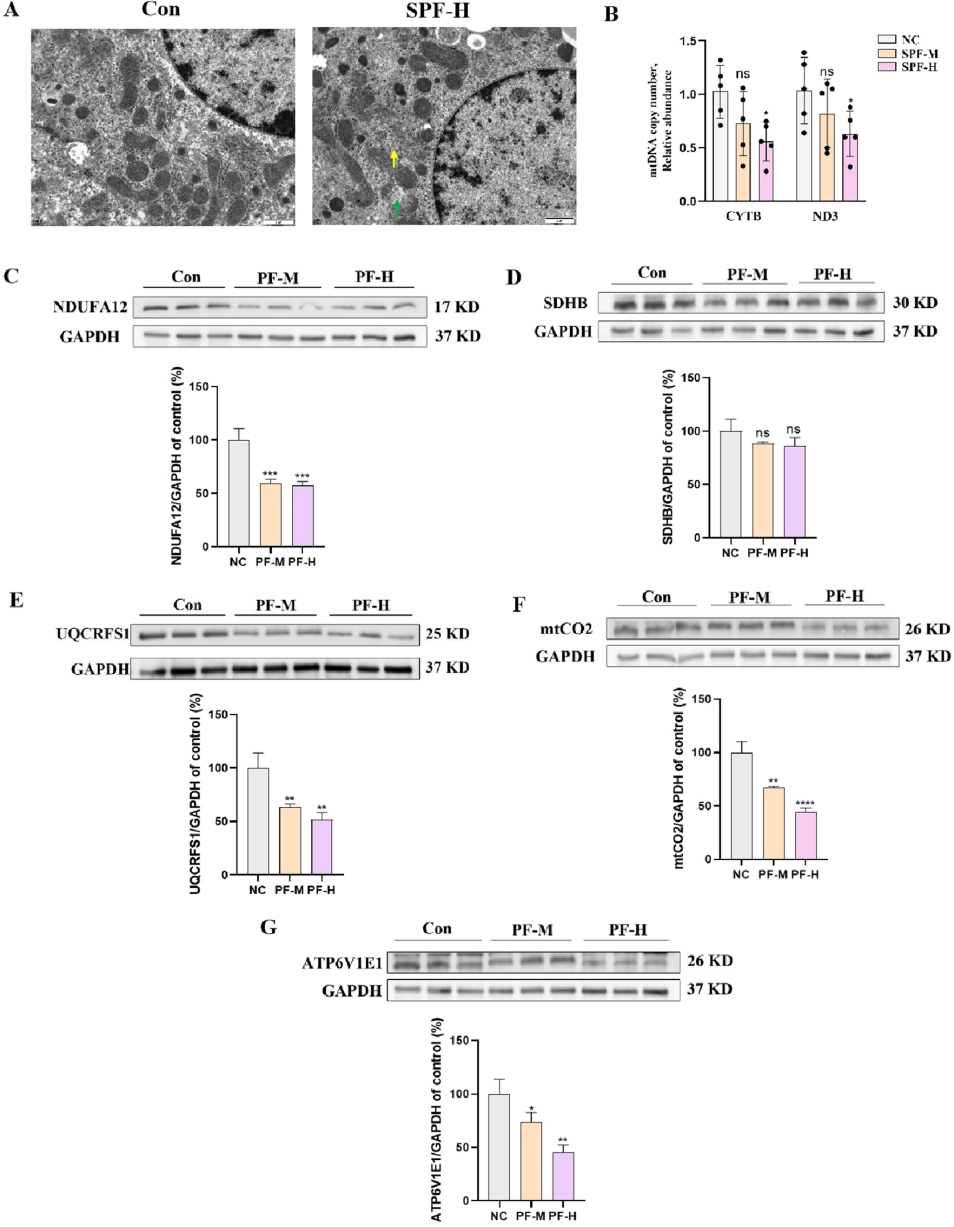
PF induced hepatic mitochondrial injury by inhibiting OXPHOS. (**A**) The ultrastructural changes of liver tissues by transmission electron microscope, scale bars = 1 μm, green and yellow arrows indicate mitochondria. (**B**) Relative abundance of mtDNA copy number. (**C–G**) Western blots detected the expression of SDHB, ATP6V1E1, UQCRFS1, mtCO2 and NDUFA12. ns, no significant; *p < 0.05, **p < 0.01, ***p < 0.001, ****p < 0.0001 versus the control group.

To validated the findings in RNA-seq and bioinformatic analysis, the ultrastructural changes of mice liver tissues were observed by transmission electron microscope. In the PF-H group, mitochondria became swollen and vacuolated, and mitochondrial cristae were broken (Green arrow in Fig. 6A). Mitochondrial bilayer membrane partially ruptured (Yellow arrow in Fig. 6A). In addition, PF lowered mtDNA copy number in a dose-dependent manner (Fig. 6B). In consistent with the results indicated by KEGG enrichment analysis, the expressions of genes in OXPHOS, including NDUFA12 (complex I), UQCRFS1 (complex III), mtCO_2_ (complex IV) and ATP6V1E1 (complex V) decreased with the treatment of PF dose-dependently (Fig. 6C,E–G), while the expressions of complex II (SDHB) showed no significant changes (Fig. 6D).

### HCS and molecular docking unveiled potential mitotoxic components in PF

**Figure 7:**
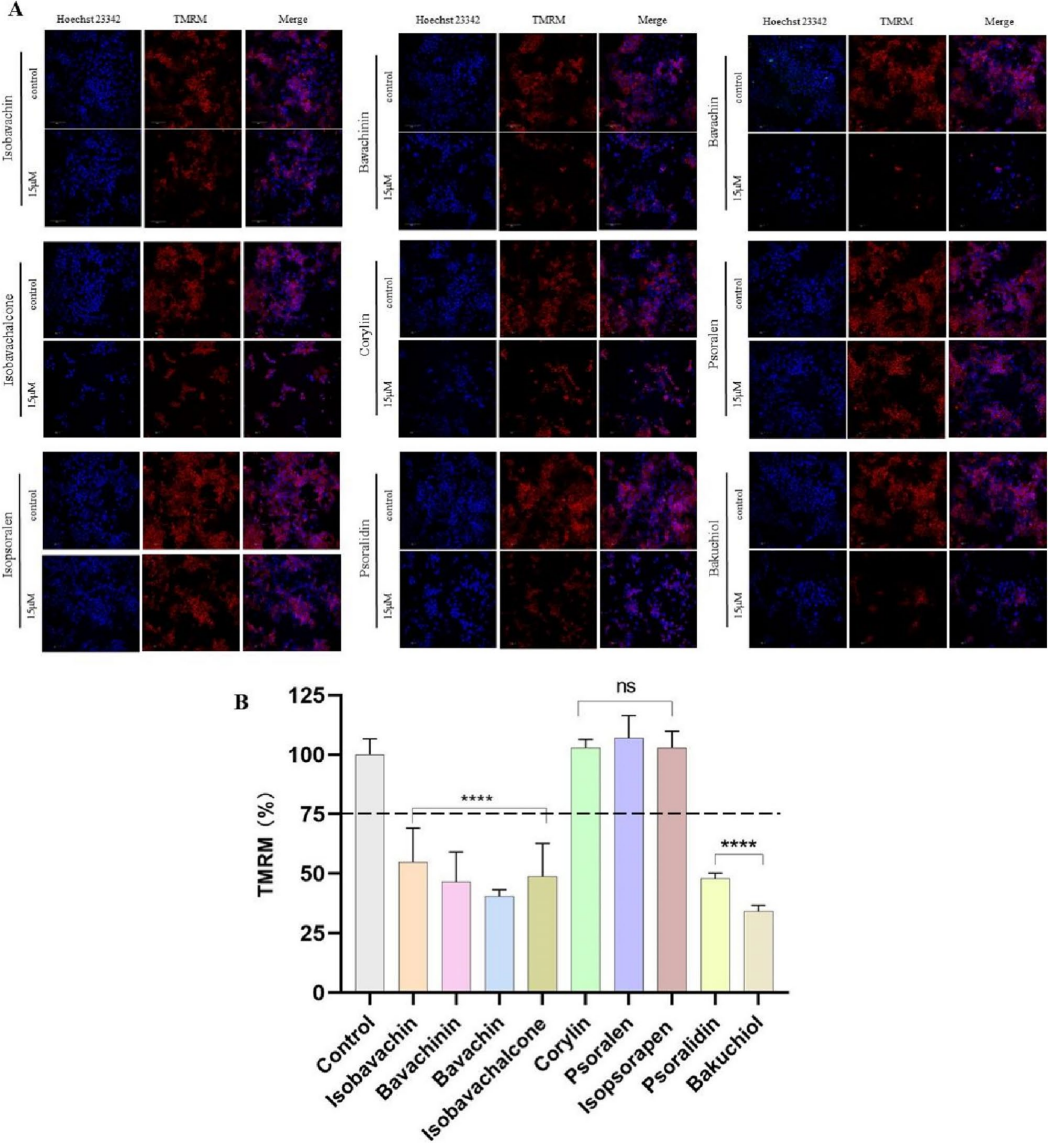
High-content analysis revealed the toxicities of 9 components in PF on MMP. (**A**) Images of high content screening. (**B**) Statistical analysis of fluorescence intensity. ns, no significant; ^****^p < 0.0001versus the control group.

**Figure 8:**
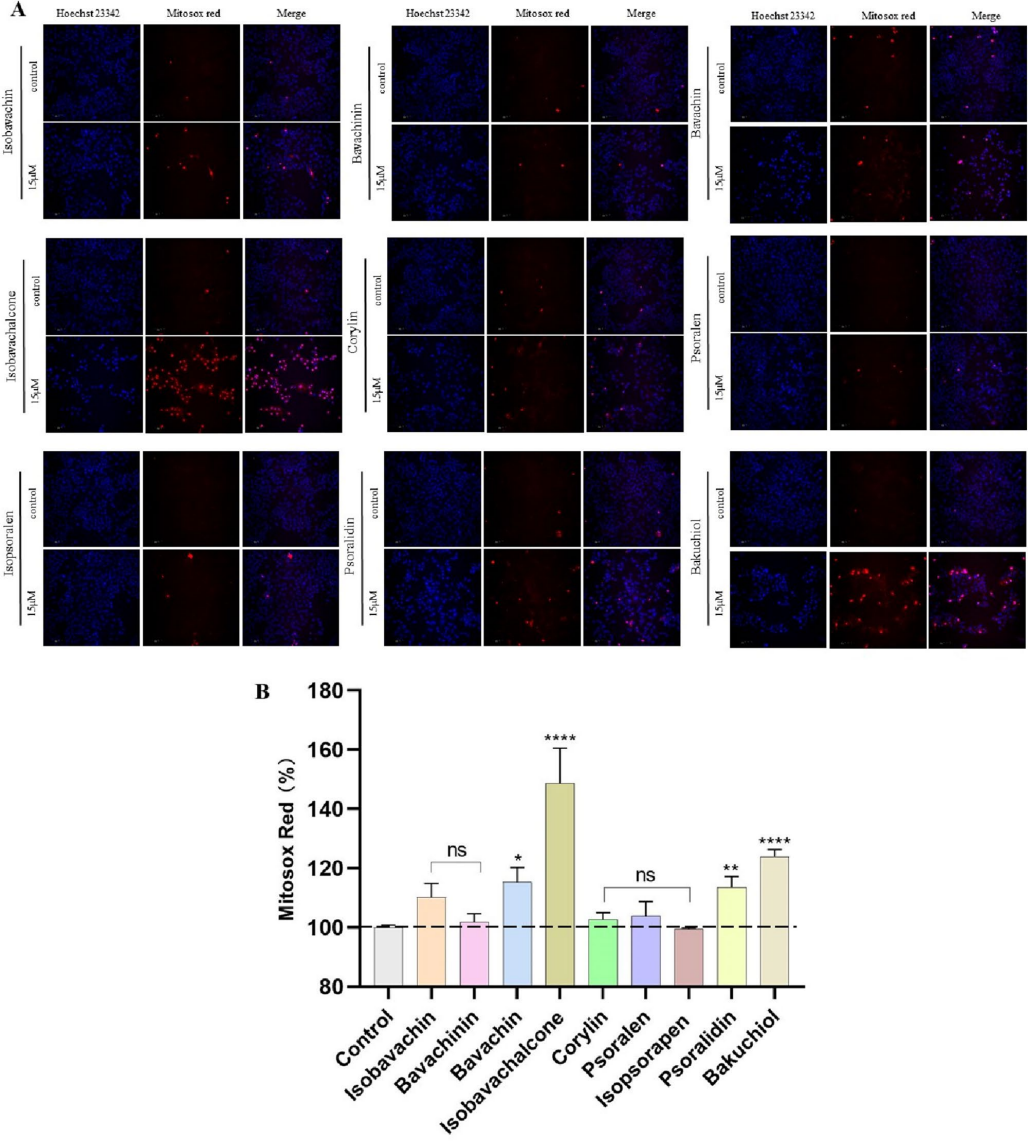
High-content analysis revealed the toxicities of 9 components in PF on mitochondrial superoxide level. (**A**) Images of high content screening. (**B**) Statistical analysis of fluorescence intensity. ns, no significant; ^*^p < 0.05, ^**^p < 0.01, ^****^p < 0.0001 versus the control group.

**Figure 9:**
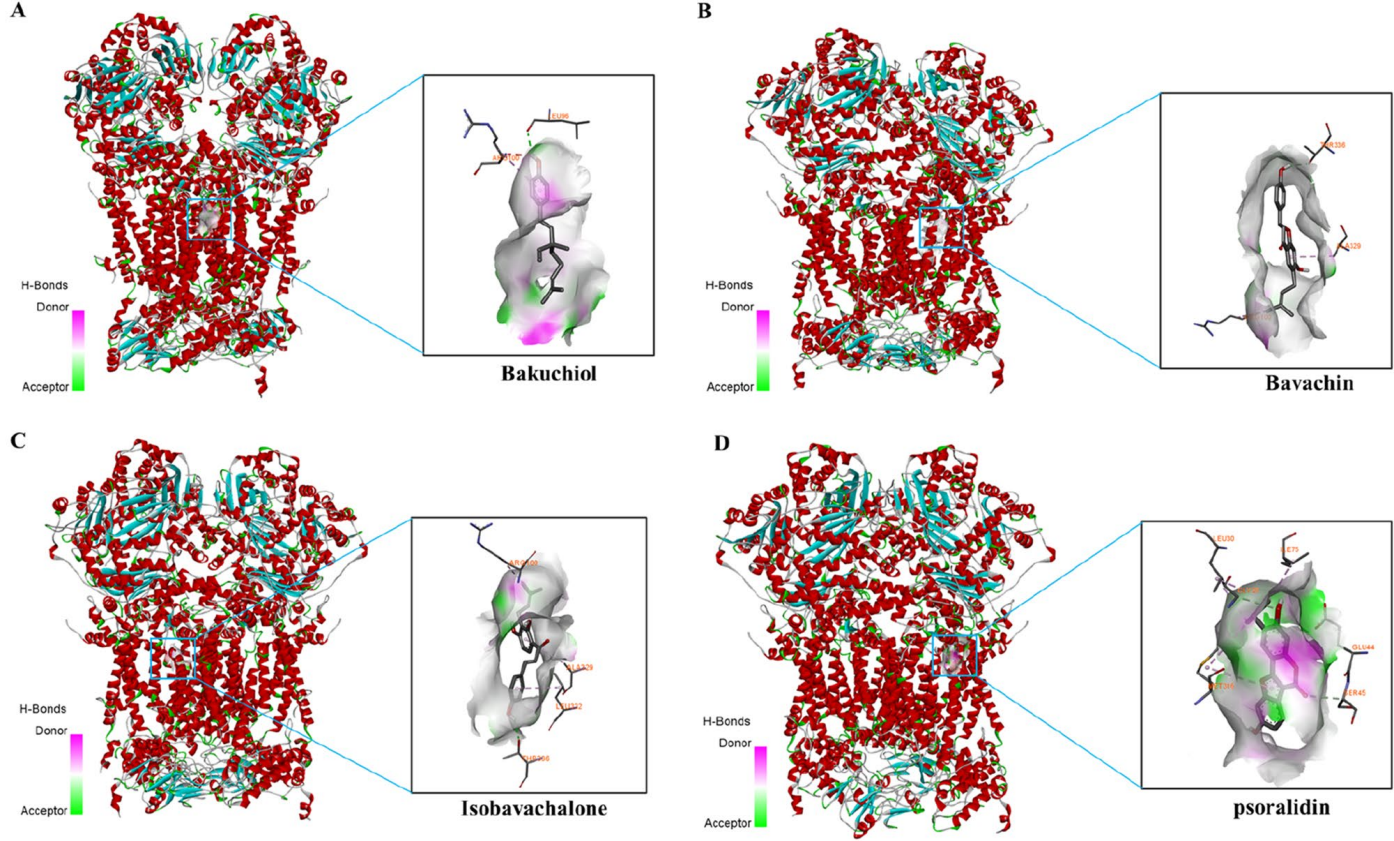
Representative molecular docking analysis of bakuchiol, bavachin, isobavachalone and psoralidin to mitochondrial respiratory complex III (PDB ID: 5XTE). (**A**) Bakuchiol (Pubchem to Cid: 5,468,522) to mitochondrial respiratory complex III. (**B**) Bavachin (Pubchem to Cid: 14,236,566) to mitochondrial respiratory complex III. (**C**) Isobavachalone (Pubchem to Cid: 5,281,255) to mitochondrial respiratory complex III. (**D**) Psoralidin (Pubchem to Cid: 5,281,806) to mitochondrial respiratory complex III.

Above all, PF-induced liver injuries were intimately associated with mitochondrial dysfunction. Therefore, we investigate the mitochondrial toxicants in PF by HCS and molecular docking. 9 representative phytochemicals from the skeleton structures of flavanone, chalcone, isoflavone, coumarin and monoterpenoid phenol were chosen. IC_50_ values were determined on AML-12 cells, showing psoralidin with the lowest IC_50_ value of 17.66 μmol/L (Supplementary Table S1). In order to compare the mitotoxicity of 9 phytochemicals with good accuracy, 15 μmol/L was selected as the testing concentration for HCS experiments. Multi-parametric mitotoxic endpoints, including cell count, MMP and mitochondrial superoxide, were evaluated by applying Hoechst 23,342, TMRM and Mitosox red as respective indicator. As shown in Figs. 6, 7 chemicals significantly decreased the fluorescence intensity of MMP indicator, except corylin, psoralen and isopsoralen. Bavachin, isobavachalcone, psoralidin and bakuchiol also prominently promoted the fluorescence intensity of Mitosox red, implicating the accumulation of mitochondrial superoxide (Fig. 8). Taken together, bavachin, isobavachalcone, psoralidin and bakuchiol exhibited the most potent mitochondrial toxicities at the same concentration. Molecular docking results further verified the binding patterns of bavachin, isobavachalcone, psoralidin and bakuchiol with mitochondrial respiratory complex III (PDB ID: 5XTE) with a strong binding affinity of − 8.9, − 8.5, − 8.3, − 7.3 kcal/mol, respectively (Fig. 9). To be specific, bavachin binds to 5XTE by conventional hydrogen bond, Pi-alkyl and alkyl interaction; Isobavachalcone binds to 5XTE by conventional hydrogen bond, Pi-alkyl and alkyl interaction; Psoralidin binds to 5XTE by conventional hydrogen bond, Pi-alkyl, Pi-anion and alkyl interaction; Bakuchiol binds to 5XTE by conventional hydrogen bond and Pi-alkyl interaction (Fig. 1). In addition, bavachin, isobavachalcone, psoralidin and bakuchiol also showed strong binding affinities with mitochondrial respiratory complex I (PDB ID: 5XTD), IV (PDB ID: 5Z62), V (PDB ID: 5WLZ) (Fig. S2–S4).

## Discussion

Mounting clinical cases reported that inappropriate administration of PF or its preparations for a long term or at a large dose could induce severe hepatic injuries with the characteristics of liver enzyme elevation, hyperbilirubinemia and histopathologic injuries^29^. Exploring potential molecular mechanism and hepatotoxic phytochemicals is beneficial for accelerating the quality control of PF and assuring its safety use. In this study, we observed that PF water extracts caused hepatic steatosis, which is similar with the finding discovered by Guo et al.^30^ in mice liver after treatment with a lower dose of PF ethanol extract. We further evaluated the influences of PF on key enzymes in the lipid metabolism. CYP7A1 is the rate-limiting enzymes that catalyzed the conversion of cholesterol to bile acids, which is strongly inhibited after PF treatment. In addition, vital enzymes participated in fatty acids β-oxidation, including CPT1, ACOX1 and ACADM, were down-regulated significantly. Therefore, the inhibitory effects of PF on lipid catabolism resulted in hepatic lipid accumulation.

The advances of omics technology provide a powerful tool to gain a systematic overview of PF-induced hepatoxicity. A total of 548 DEGs were found in PF-treated mice livers, among which 220 upregulated genes and 328 downregulated genes. KEGG analysis indicated DEGs mainly enriched in OXPHOS. Mitochondria functions as the powerhouse and metabolic hub in cells. Metabolites derived from catabolism of glucose, fatty acids and amino acids are utilized in TCA cycle to supply substrates that enter the ETC for OXPHOS. Through orchestrating complex I–V in ETC, mitochondria generated ATP with the process of substrate degradation and oxygen consumption^31^. Impaired OXPHOS could promote lipid metabolism disorder and hepatic steatosis. Various drugs-mediated liver injuries have been proved to be attributed to its direct or indirect disruption of mitochondria^32,33^. In the study, mitochondrial ultrastructure damage was observed in mice liver after the treatment with PF water extracts. Dose-dependent mtDNA depletion was also detected, which is usually associated with OXPHOS deficiency. Western blot results indicated PF significantly downregulated the gene expressions of complex I, complex III, complex IV and complex V. Both transcriptomic analysis and experimental results validated OXPHOS deficiency is the main cause of PF-induced hepatic injuries.

Hinted by above findings, the hepatotoxic phytochemicals were screened by its effects on mitochondrial function. As a compound library, PF contained a large amount of phytochemicals with various kinds of skeleton structure. In order to gain a comprehensive understanding on the relationship between skeleton structure and hepatotoxicity, 9 representative compounds with 5 different skeleton structure were selected. HCS is an automated image analysis system of cellular assays with simultaneous detection of multiple phenotypic parameters^34^. As an efficient high-throughput fluorescence microscopy technology, HCS has been a powerful tool in the areas of toxicology and drug discovery^35^. According to the IC_50_ values, we explored the hepatotoxicity of 9 components at the same concentration of 15 μmol/L by utilizing specific fluorescent probe of MMP and mitochondrial superoxide. Indicated by HCS analysis, bavachin, isobavachalcone, psoralidin and bakuchiol exhibited the strongest mitochondrial toxicities. Previous studies on the contents of phytochemicals in PF showed the level of bakuchiol and isobavachalcone were higher than other compounds. Therefore, we preliminarily speculated bakuchiol and isobavachalcone are the main toxic components in PF. Further in vivo validation and deep mechanism studies on the hepatotoxicities of bakuchiol and isobavachalcone are necessarily needed.

## Conclusion

In summary, PF promoted hepatic lipid accumulation by inhibiting fatty acids β-oxidation and conversion of cholesterol to bile acids. RNA-seq, bioinformatic analysis and experimental results validated PF down-regulated the expression of respiratory chain complex, leading to mitochondrial structure remodeling and dysfunction. Hinted by the molecular mechanism, hepatotoxic constituents were screened by HCS on the basis of mitotoxic endpoints. Further validation by molecular docking indicated bavachin, isobavachalcone, psoralidin and bakuchiol as contributors to PF-induced hepatotoxicity. This study provided a systematic approach to investigate mechanism and phytochemicals in PF-induced liver injuries from the aspect of mitochondrial dysfunction.

### Supplementary Information

Supplementary Information.

## Data Availability

The RNA-seq datasets generated in this study have been submitted to National Center for Biotechnology Information (NCBI) under Accession No. PRJNA1035563.
