# Supplementary material for: Exploring the mechanism and phytochemicals in Psoraleae Fructus-induced hepatotoxicity based on RNA-seq, in vitro screening and molecular docking
Source: Sci Rep. 2024 Jan 19;14:1696. doi: 10.1038/s41598-023-50454-0 (PMC10799058; doi:10.1038/s41598-023-50454-0)

Table 1 The IC_{50} values of 7 compounds in PF.

Compounds	IC_{50} toward AML12 cells (μmol/L)	R-squared
Psoralidin	17.66	0.9474
Isobavachalcone	28.88	0.9792
Bavachin	33.25	0.9158
Isobavachin	84.61	0.911
Bavachinin	60.21	0.9604
Corylin	34.39	0.8331
Bakuchiol	34.95	0.9233

To uncover the potential hepatotoxic components, CCK-8 assay was performed to evaluate the toxic effects of 9 chemicals in PF on AML-12 cells. With the treatment of individual chemical for 24 h, the IC_{50} values of psoralidin, isobavachalcone, bavachin, isobavachin, bavachinin, corylin and bakuchiol were determined as 17.66, 28.88, 33.25, 84.61, 60.21, 34.39 and 34.95 μmol/L to AML-12 cells, respectively (Table 2). Psoralen and isopsoralen poorly dissolved in DMEM/F12 culture medium containing 1‰ DMSO and exhibited no obvious toxicity until reaching their maxima solubility (600 μmol/L for psoralen and 800 μmol/L for isopsoralen), which led to the failure in the determination of their IC_{50} values.

Method:

AML-12 cells were seeded into 96-well plates for 24 h until they visibly reached confluence. Then, AML-12 cells were treated with different concentrations of the nine chemical compounds in PF for 24 h. The multiple concentrations of each compound

were as follows: isobavachin (0, 10, 20, 30, 40, 50, 60, 70, 80, 90 $\mu mol \cdot L^{-1}$), bavachinin and bavachin (0, 10, 20, 30, 40, 50, 60, 70, 80, 90, 100 $\mu mol \cdot L^{-1}$), isobavachalcone and corylin (0, 10, 20, 30, 40, 50, 60 $\mu mol \cdot L^{-1}$), psoralen and isopsoralen (0, 100, 200, 300, 400, 500, 600, 700, 800 $\mu mol \cdot L^{-1}$), psoralidin (0, 10, 20, 30, 40, 50, 60 $\mu mol \cdot L^{-1}$), bakuchiol (0, 10, 20, 30, 40, 50 $\mu mol \cdot L^{-1}$); Five replicate wells were set up for each group. After treatment, AML-12 cells were washed with PBS buffer gently and incubated with DMEM/F12 medium containing 10% CCK-8 solution for 0.5-2 h. OD value was measured with live cell imaging system (Cytation 5, BioTek, USA) at 450 nm.

Cell viability (%) = [(OD value of the experimental group − OD value of the blank group)/ (OD value of the control group − OD value of the blank group)] × 100%.

Figure S1. Binding types of bavachin, isobavachalcone, psoralidin and bakuchiol to 5XTE. (A) Binding types of bavachin to 5XTE; (B) Binding types of isobavachalcone to 5XTE; (C) Binding types of psoralidin to 5XTE; (D) Binding types of bakuchiol to 5XTE.

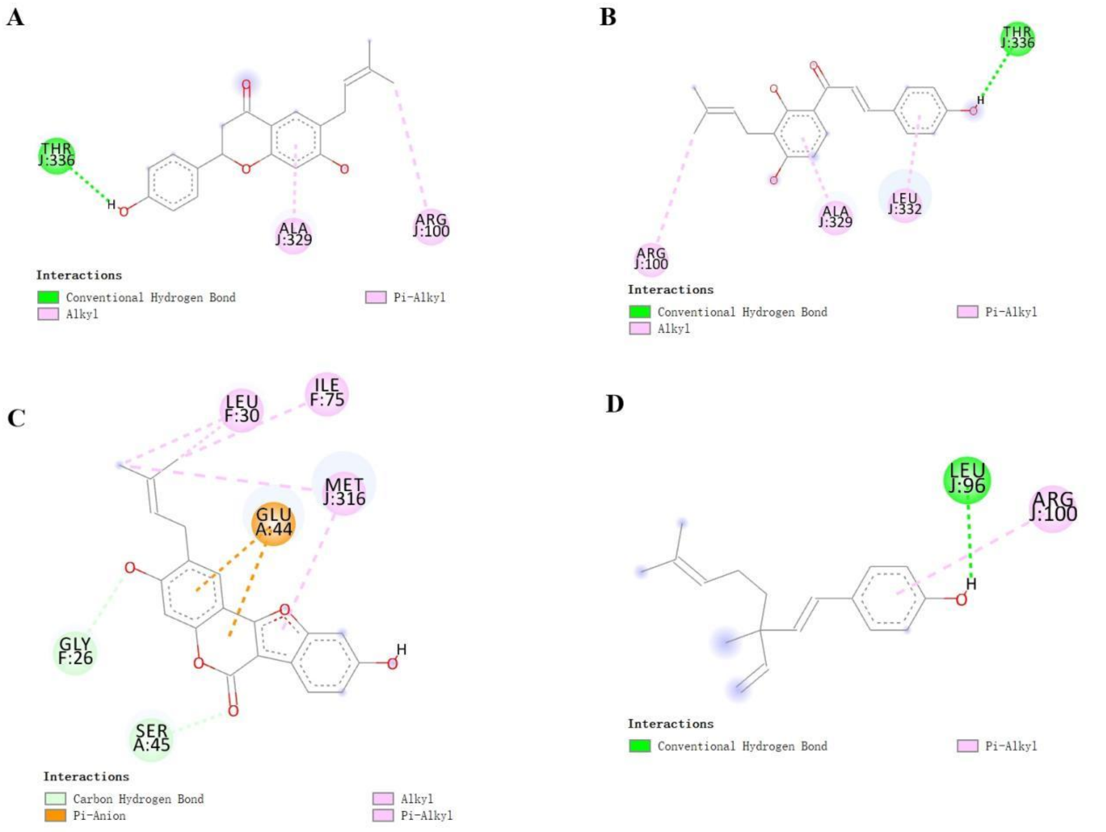

Figure S2. Representative molecular docking analysis of bakuchiol, bavachin, isobavachalone and psoralidin to mitochondrial respiratory complex I (PDB ID: 5XTD). (A) Bakuchiol (Pubchem to Cid: 5468522) binds to mitochondrial respiratory complex I with a strong binding affinity of -5.8 kcal/mol. (B) Bavachin (Pubchem to Cid: 14236566) binds to mitochondrial respiratory complex I with a strong binding affinity of -7.3 kcal/mol. (C) Isobavachalone (Pubchem to Cid: 5281255) binds to mitochondrial respiratory complex I with a strong binding affinity of -7.1 kcal/mol. (D) Psoralidin (Pubchem to Cid: 5281806) binds to mitochondrial respiratory complex I with a strong binding affinity of -7.8 kcal/mol.

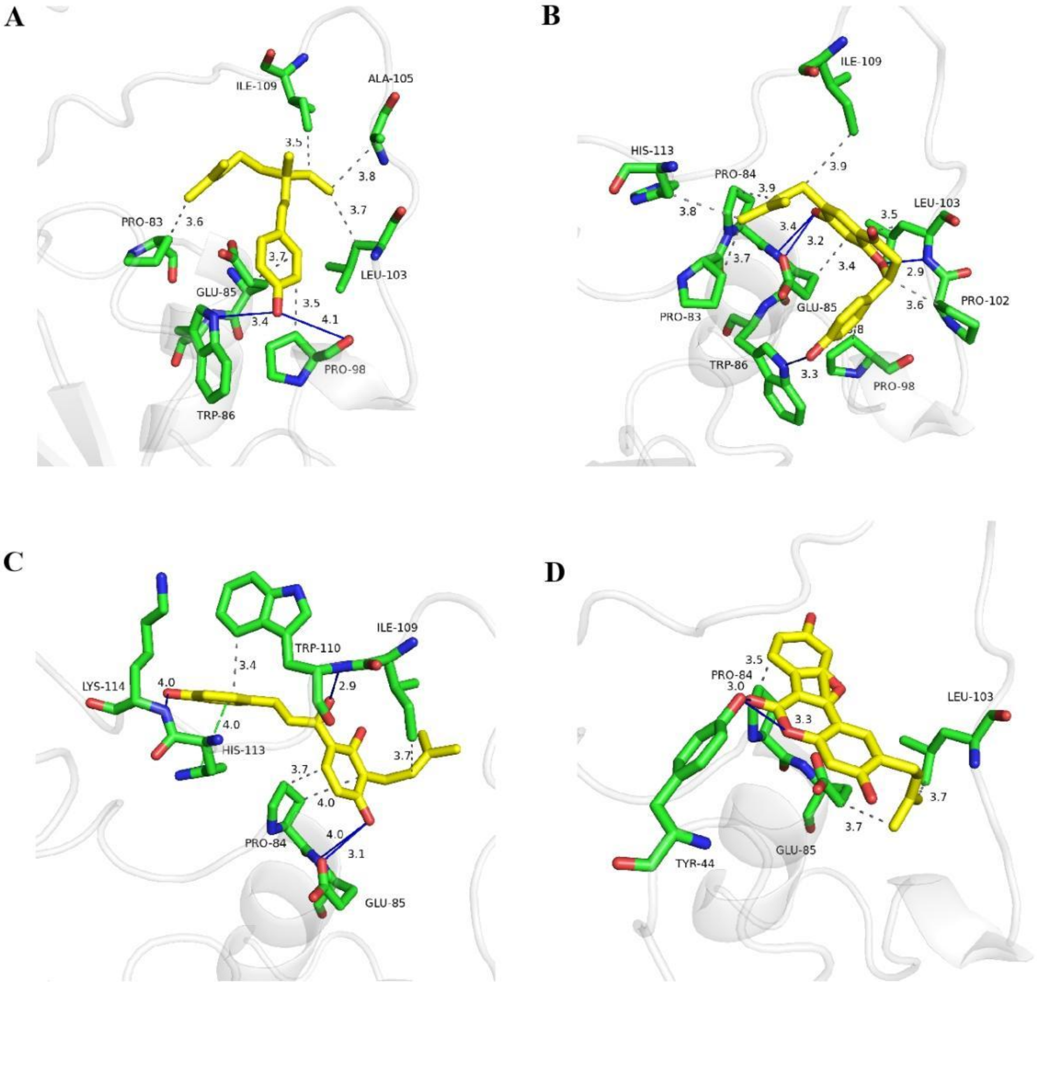

Figure S3. Representative molecular docking analysis of bakuchiol, bavachin, isobavachalone and psoralidin to mitochondrial respiratory complex IV (PDB ID: 5Z62). (A) Bakuchiol (Pubchem to Cid: 5468522) binds to mitochondrial respiratory complex IV with a strong binding affinity of -5.9 kcal/mol. (B) Bavachin (Pubchem to Cid: 14236566) binds to mitochondrial respiratory complex IV with a strong binding affinity of -6.9 kcal/mol. (C) Isobavachalone (Pubchem to Cid: 5281255) binds to mitochondrial respiratory complex IV with a strong binding affinity of -7.9 kcal/mol. (D) Psoralidin (Pubchem to Cid: 5281806) binds to mitochondrial respiratory complex IV with a strong binding affinity of -7.6 kcal/mol.

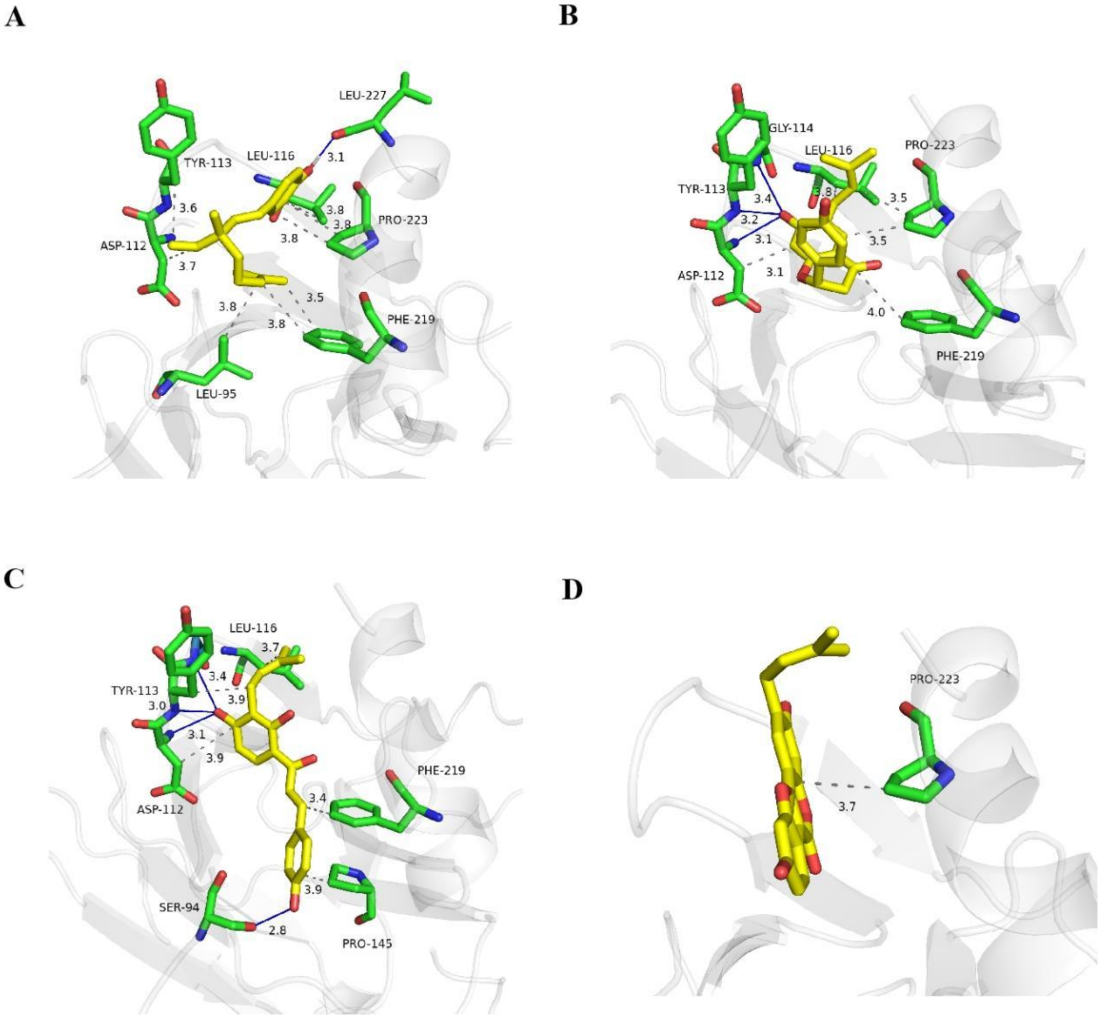

Figure S4. Representative molecular docking analysis of bakuchiol, bavachin, isobavachalone and psoralidin to mitochondrial respiratory complex V (PDB ID: 5WLZ). (A) Bakuchiol (Pubchem to Cid: 5468522) binds to mitochondrial respiratory complex V with a strong binding affinity of -5.2 kcal/mol. (B) Bavachin (Pubchem to Cid: 14236566) binds to mitochondrial respiratory complex V with a strong binding affinity of -6.9 kcal/mol. (C) Isobavachalone (Pubchem to Cid: 5281255) binds to mitochondrial respiratory complex V with a strong binding affinity of -6.7 kcal/mol. (D) Psoralidin (Pubchem to Cid: 5281806) binds to mitochondrial respiratory complex V with a strong binding affinity of -6.8 kcal/mol.

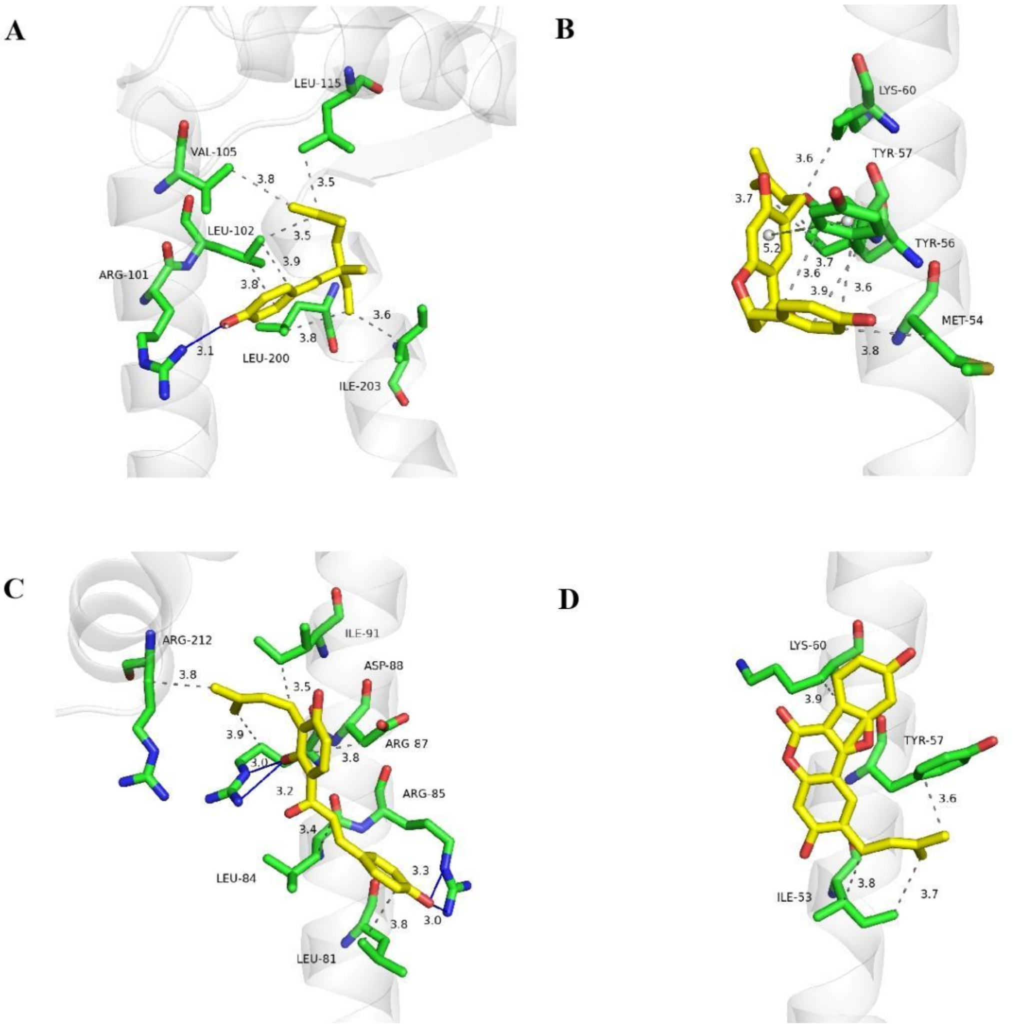

Original western blots corresponding to Figure 3 and Figure 6. Each PVDF membrane was only cropped into two parts. One part is incubated with the antibody of house-keeping gene, the other is incubated with the antibody of target gene.

1. Original blots for CPT1

CPT1

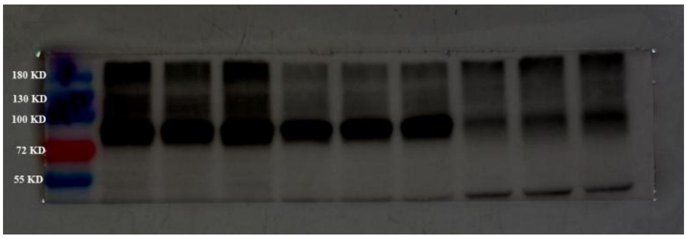

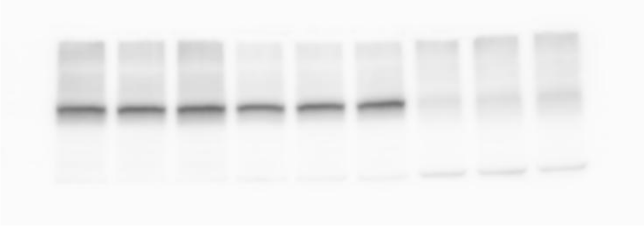

GAPDH

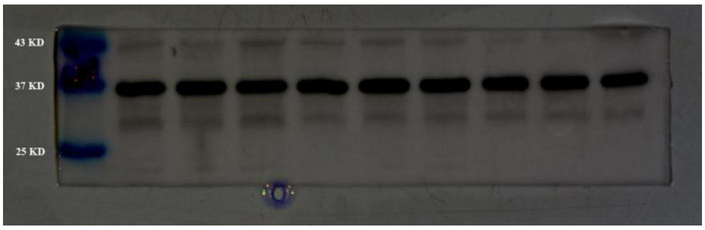

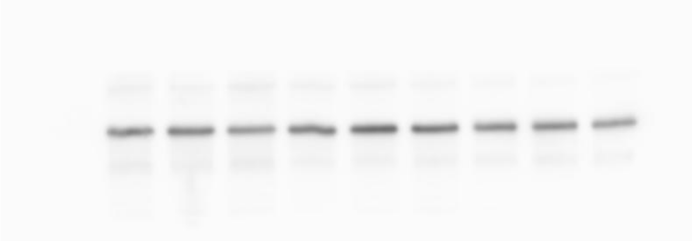

2. Original blots for CYP7A1

CYP7A1

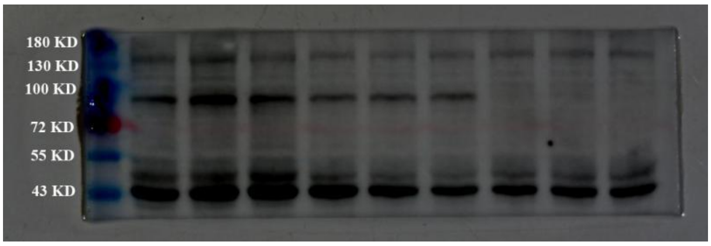

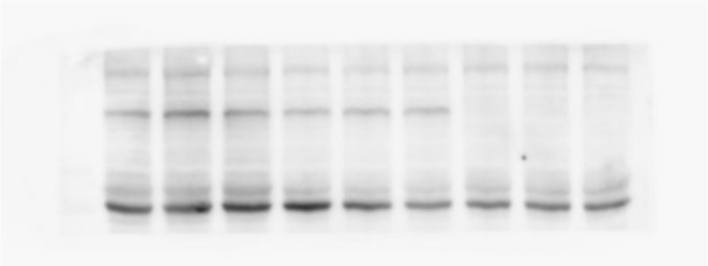

GAPDH

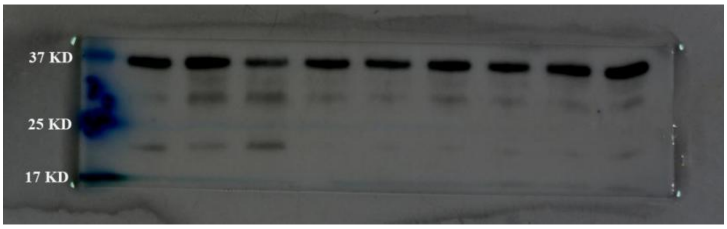

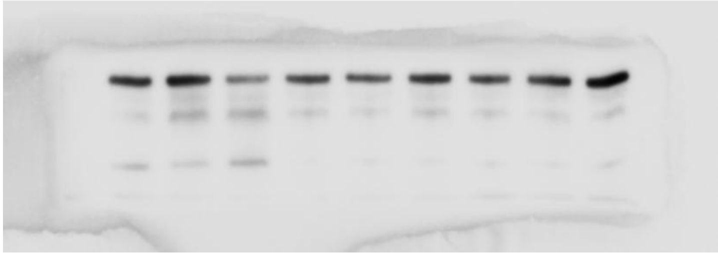

3. Original blots for ACOX1

ACOX1

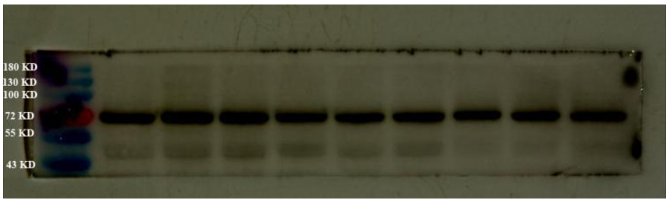

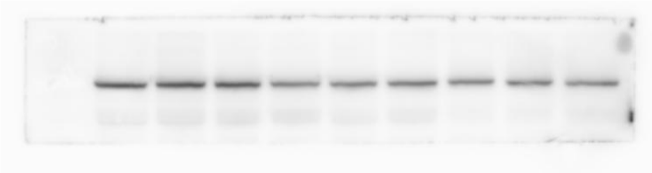

GAPDH

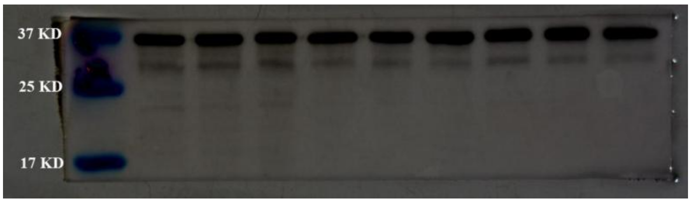

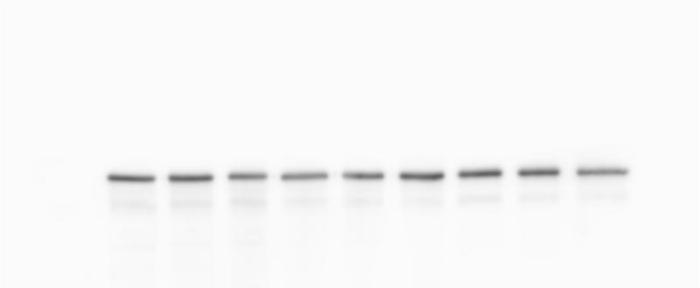

4. Original blots for ACADM

1）ACADM

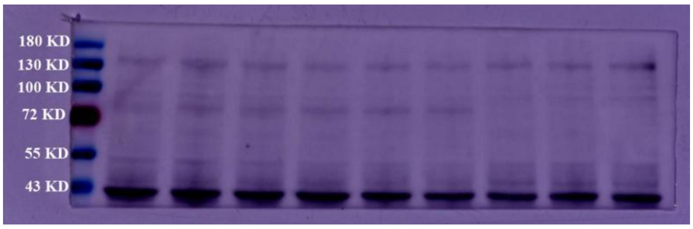

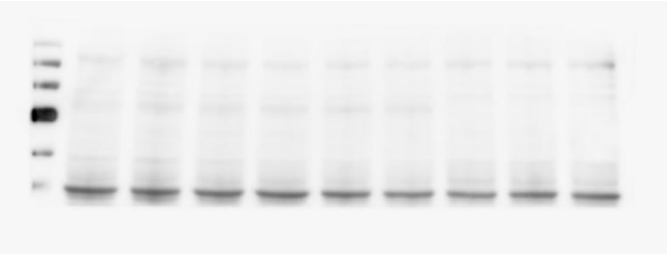

GAPDH

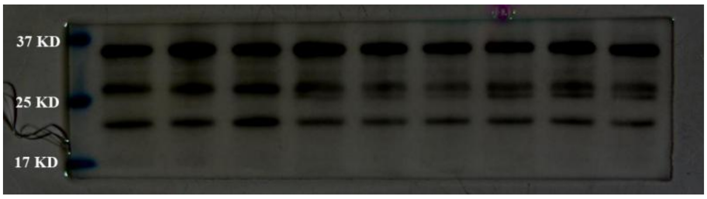

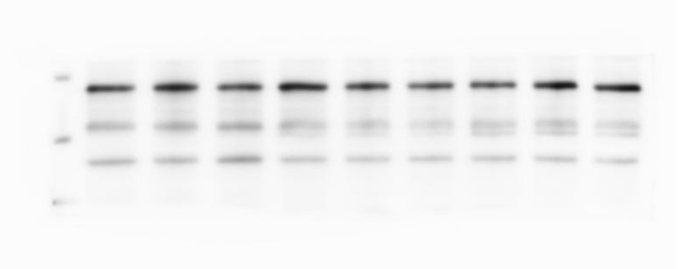

5. Original blots for SDHB

SDHB

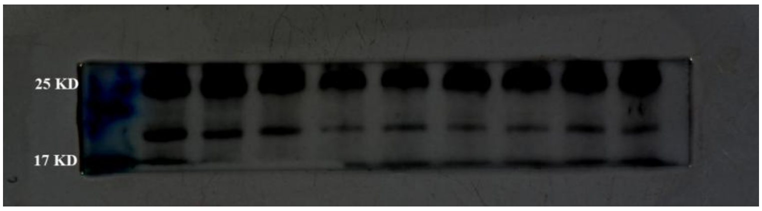

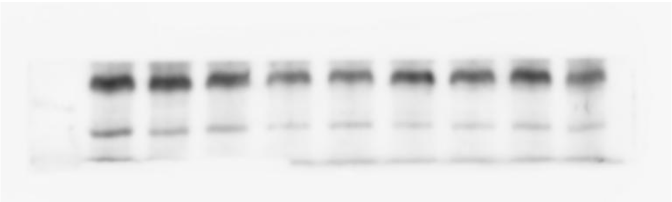

GAPDH

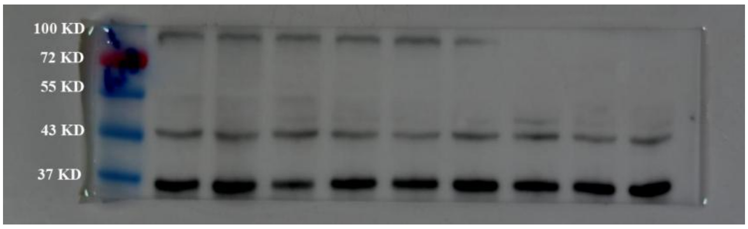

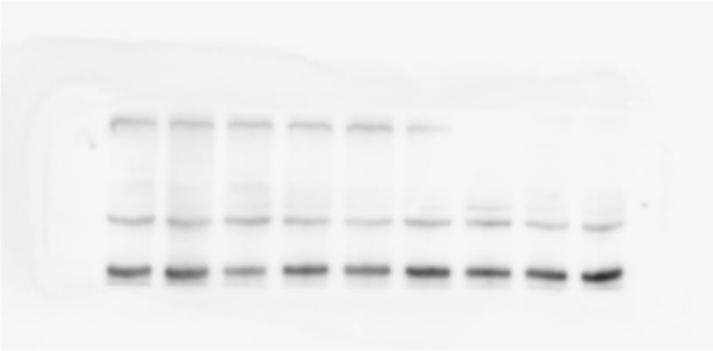

6. Original blots for ATP6V1E1

ATP6V1E1

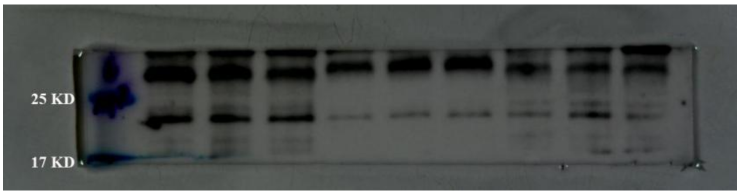

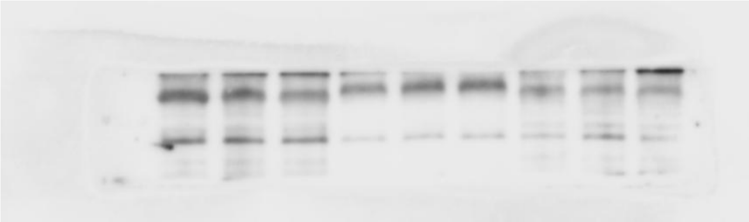

GAPDH

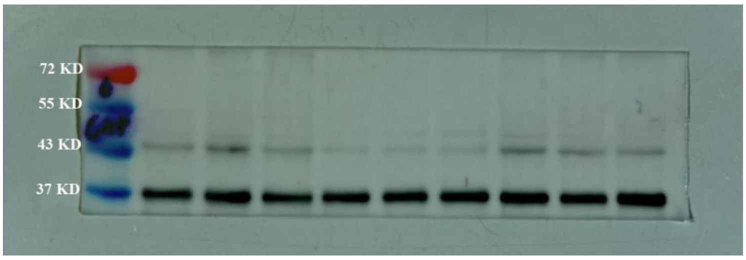

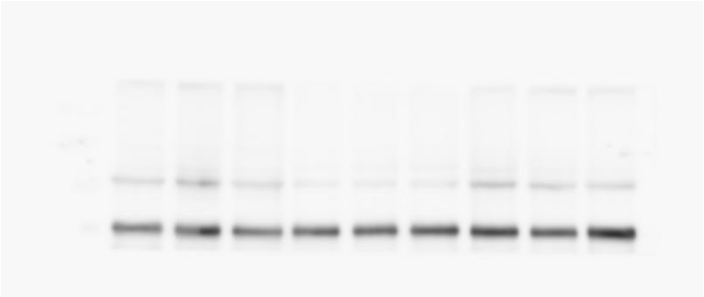

7. Original blots for UQCRFS1

UQCRFS1

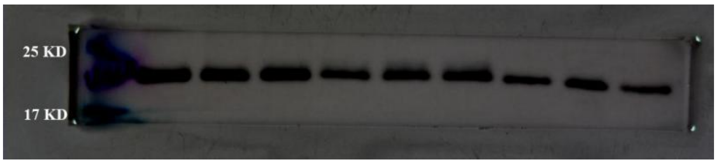

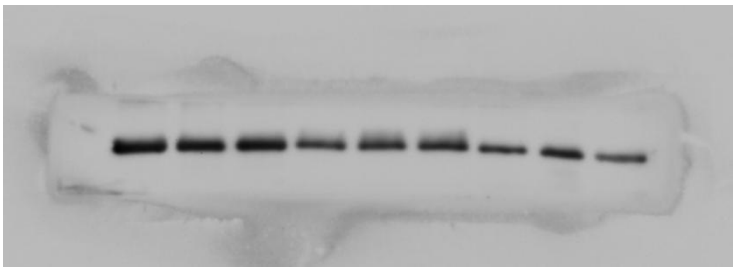

GAPDH

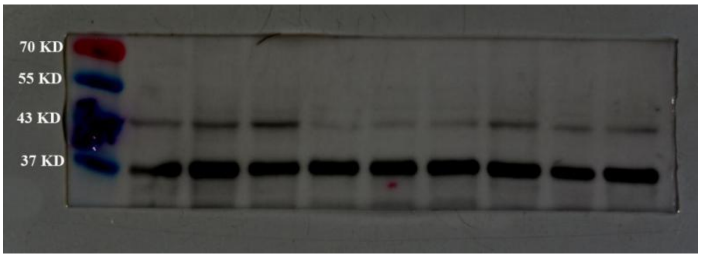

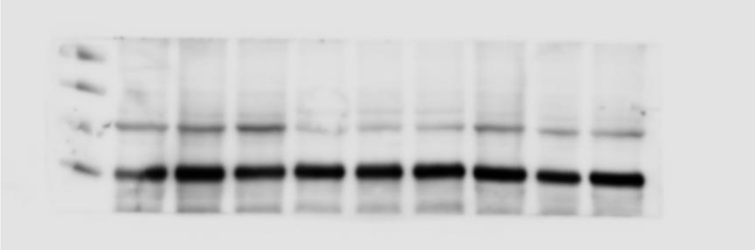

8. Original blots for mtCO2

mtCO2

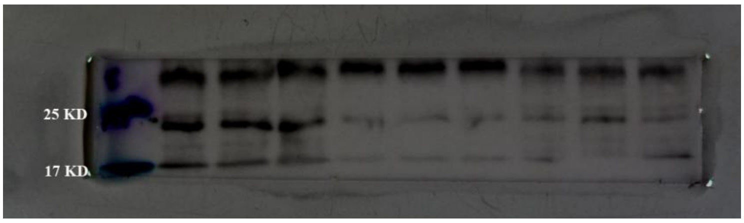

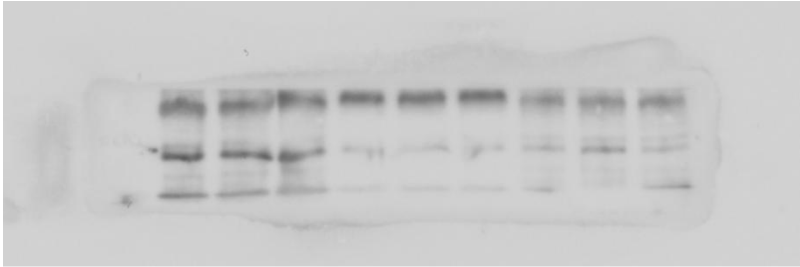

GAPDH

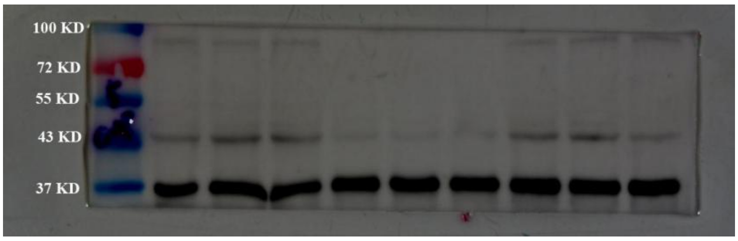

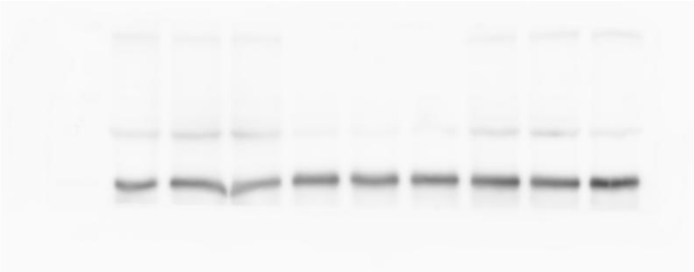

9. Original blots for NDUFA12

NDUFA12

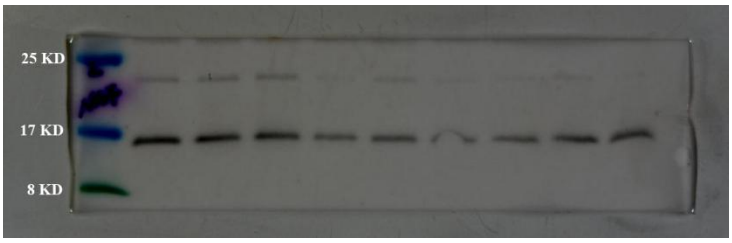

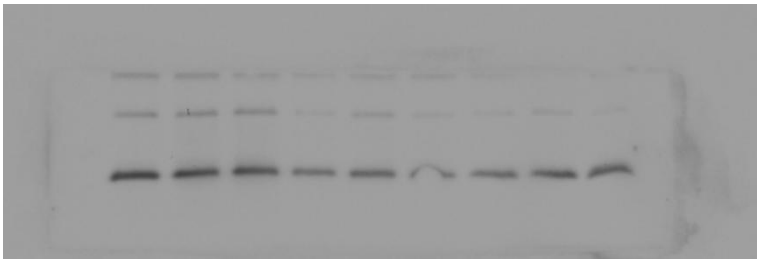

GAPDH

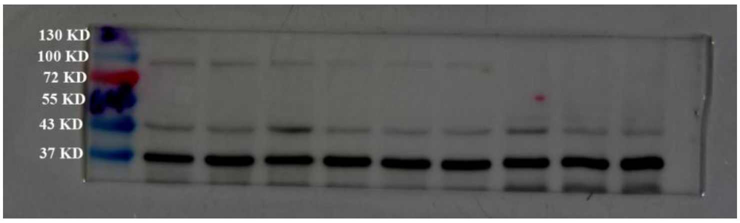

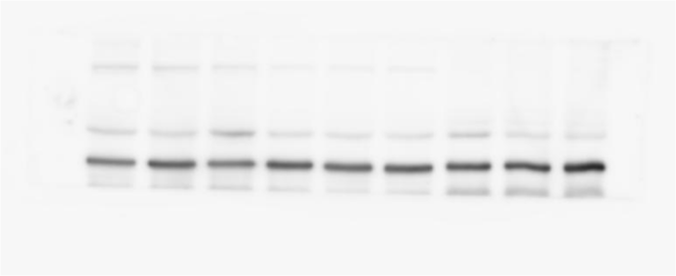

Supplement: Supplementary file 1 — Supplementary Information. [file 41598_2023_50454_MOESM1_ESM.pdf]
